# Beneficial effect of *Punica granatum* peel extract on murine malaria-induced spleen injury

**DOI:** 10.1186/s12906-016-1207-9

**Published:** 2016-07-16

**Authors:** Murad A. Mubaraki, Taghreed A. Hafiz, Mohamed A. Dkhil, Saleh Al-Quraishy

**Affiliations:** Clinical Laboratory Sciences Department, College of Applied Medical Sciences, King Saud University, Riyadh, Saudi Arabia; Department of Zoology, College of Science, King Saud University, Riyadh, Saudi Arabia; Department of Zoology and Entomology, Faculty of Science, Helwan University, Helwan, Egypt

**Keywords:** Pomegranate, *Plasmodium chabaudi*, Oxidative stress, Apoptosis

## Abstract

**Background:**

Multiple drug-resistant malaria parasites have been widely detected, which has encouraged research studies focused on discovering alternative therapies. Medicinal plants such as pomegranate, *Punica granatum*, have been proven to exhibit antiprotozoal effects and therefore, we examined its effects on murine malaria-induced splenic injury and oxidative stress in this study.

**Methods:**

Mice were divided into three groups, a vehicle control and two groups that were infected with 10^6^*Plasmodium chabaudi-*parasitized red blood cells (RBCs). The third group was gavaged with 100 μL of 300 mg/kg pomegranate peel extract for 6 days. All mice were euthanized on day 6 post-infection.

**Results:**

The results revealed the potential antimalarial, antioxidant, and anti-inflammatory effects of pomegranate. Furthermore, pomegranate peel extracts significantly reduced parasitemia and spleen index of the treated mice compared to the untreated group. Additionally, the spleen histology score supported the findings by showing better amelioration in the pomegranate-treated mice than in the untreated mice. Concomitantly, the spleen capsule thickness showed clear evidence of splenomegaly in the untreated mice, as evidenced by the reduced spleen capsule. However, pomegranate peel extract exhibited a remarkable restorative effect on the spleen capsules of the treated mice. Moreover, the extract significantly reduced the expression levels of the proinflammatory cytokines interleukin (IL)-1β, tumor necrosis factor (TNF)-α, and interferon (IFN)-γ as well as inducible nitric oxide synthase (iNOS). Moreover, our study showed that pomegranate extract profoundly affected oxidative stress levels by reducing the oxidant molecules, nitric oxide (NO) and malondialdehyde (MDA).

**Conclusion:**

This study showed that pomegranate clearly induced antimalarial activity in the host by attenuating inflammatory and oxidative stress responses. Furthermore, pomegranate enhanced the innate immune responses and, therefore, could serve an alternative therapy to control clinical malaria episodes and may protect against malaria infection.

## Background

Malaria is a serious ancient infectious disease that affects millions of people yearly [1]. It is characterized by recurrent febrile episodes known as malaria paroxysm, which concurs with the rupture of schizont-infected red blood cells (RBCs). This clinical symptom is associated with enlargement of the spleen, which acts to rapidly remove parasitized RBCs (pRBCs), particularly after treatment [2]. The spleen is believed to have a vital role in combating malaria infections by activating the immune response and destroying the pRBC [3, 4]. This report is supported by a study showing the protective role of the spleen in mice infected with *Plasmodium chabuadi* [5]. However, although the spleen acts as an effector for malaria clearance, it is less efficient under conditions of high parasitemia infection levels. Malaria induces inappropriate or excessive immune responses by inducing proinflammatory cytokines including interleukin (IL)-1β, IL-6, tumor necrosis factor (TNF)-α, interferon (IFN)-γ, and inducible nitric oxide synthase (iNOS). Therefore, most malaria pathogenic processes are surmounted by the immune system of the body, which acts to eliminate malaria parasites [6, 7].

The defense mechanism of the host against the parasites is mediated by specialized cells. Various oxidant molecules such as malondialdehyde (MDA) and NO, which are generated in the body, play an important role in this defense mechanism that adversely affects the viability of the parasites [8]. MDA is a marker of free radical activity and lipid peroxidation, which contribute to cellular injury. NO is a molecule with free radical characteristics, and it is thought to be a mediator of the malaria infection process [9].

The rodent malaria parasite, *P. chabaudi*, is a convenient model to study the role of the spleen in malaria infections because it exhibits many of the pathological and immunological features of the most dangerous human malaria parasite, *P. falciparum* [10]. *P. chabaudi* causes a chronic, non-lethal infection by invading the RBC at all stages [11]. This rodent model of malaria has been used and still serves as an excellent model for enhancing the understanding of human malaria infections and identifying new drug targets [12].

The emergence of multiple drug-resistant malaria parasites has initiated a search for new antimalarial agents from a variety of sources. Different parts of the *Punica granatum* L., tree (pomegranate), including the peel, seeds, and bark, have been used for centuries as a distinctive remedy in traditional medicine to ameliorate a range of diseases [13]. Studies have shown that pomegranate peel extract (PPE) exhibits antibacterial, antifungal [14], antiprotozoal [15], antihelminthic, and antioxidant activities [16]. Recently, Hafiz et al. [17] reported that pomegranate peel has a protective role in murine malaria-induced hepatic injury. Therefore, the present study aimed to examine the effect of *P. granatum* treatment on murine malaria-induced splenic damage, apoptosis, and oxidative stress.

## Methods

### PPE preparation

The pomegranate fruits were purchased from a local market, and the samples were authenticated by Dr. Jacob Thomas (Botany Department, College of Science, King Saud University, Saudi Arabia). The PPE was prepared in accordance with the method previously described by Abdel Moneim [18] with slight modifications. In brief, the pomegranate peels were air-dried, powdered, extracted with 70 % methanol at 4 °C for 24 h. The obtained extract was concentrated under reduced pressure (bath temperature 50 °C) and dried using a vacuum evaporator. The filtrate was subsequently dissolved in distilled water prior to being used for the entire study.

### Animals

Thirty male Swiss albino mice, aged 10–12 weeks, were housed, bred under specified conditions, and provided a standard diet and water *ad libitum*.

### Infection of mice

The blood stages of the *P. chabaudi* parasite were passaged in Swiss albino mice on a weekly basis. The experimental animals were injected with 10^6^*P. chabaudi*-parasitized red blood cells (pRBCs). Then Giemsa-stained blood smears were prepared, and the pRBCs and total RBCs were counted to evaluate the level of parasitemia [19].

### Experimental design

The animals were divided into three groups consisting of an uninfected vehicle control and two groups that were infected with 10^6^*P. chabaudi-*pRBCs. The third group was gavaged with 100 μL of 300 mg/kg PPE for 6 days [16]. All the mice were euthanized on day 6 post-infection (p.i.).

### Histology of spleen

Samples of the spleen tissue were formalin-fixed at room temperature overnight, embedded in paraffin, 5-μm sections were cut, and then stained with hematoxylin and eosin. The enlargement of white pulp areas of the spleen segments was scored using the following scale: (0, absent; 1, slight; 2, moderate; and 3, pronounced). In addition, the scoring of the increased numbers of apoptotic cells, macrophages, necrotic cells, and the presence of pigments was based on the following scale: (0, absent and 1, present). The final score of each tissue sample was the mean score of the high magnification microscopy fields of five different sections.

### Apoptotic changes in spleen

The paraffin-embedded spleen sections were deparaffinized, rehydrated in graded ethanol solutions, and then a terminal deoxynucleotidyl transferase (TdT) dUTP nick-end labeling (TUNEL) assay for apoptosis was performed according to the manufacturer’s protocol (GenScript, Piscataway, NJ, USA). The sections were counterstained with hematoxylin [20].

### Biochemical analysis

A 50 % (*w/v*) spleen homogenate was prepared as follows. A sample of the spleen tissue was weighed and homogenized immediately in ice-cold medium containing 50 mM Tris-hydrochloride (HCl) and 300 mM sucrose. The homogenate was then centrifuged at 500 × *g* for 10 min at 4 °C. The supernatant was diluted with the Tris-sucrose buffer to a final concentration of 10 % and was then used for the biochemical analysis.

The catalase activity of the spleen homogenate was assayed by using the method of Aebi [21]. In this assay, catalase reacts with a known quantity of hydrogen peroxide (H_2_O_2_) and the reaction is stopped after exactly 1 min with a catalase inhibitor. The remaining H_2_O_2_ then reacts with 3,5-dichloro-2- hydroxybenzene sulfonic acid and 4-aminophenazone in the presence of horseradish peroxidase to form a chromophore with a color intensity that is inversely proportional to the amount of catalase in the original sample, and is measured at 240 nm.

The lipid peroxidation level of the spleen homogenate was determined according to the method of Ohkawa et al. [22] using 1 mL each of 10 % trichloroacetic acid and 0.67 % thiobarbituric acid 0.67 %, followed by heating in a boiling water bath for 30 min. The thiobarbituric acid reactive substances were determined by reading the absorbance of the reaction solution at 535 nm and were expressed as MDA equivalents formed.

The NO assay of the spleen homogenate was performed according to the method of Berkels et al. [23]. In an acid medium in the presence of nitrite, the generated nitrous acid diazotized the sulfanilamide, which was then coupled with N-(1-naphthyl) ethylenediamine. The resulting azo dye had a bright reddish-purple color that was measured at 540 nm.

### Quantitative real-time polymerase chain reaction (qPCR)

The spleen tissue samples were aseptically removed, rapidly frozen, and stored in liquid nitrogen until they were used. The total RNA was isolated using Trizol reagent (Invitrogen), the RNA samples were subsequently treated with DNase (Applied Biosystems, Darmstadt, Germany) for at least 1 h, and were then transcribed into cDNA using a reverse transcription kit (Qiagen, Hilden, Germany), following the manufacturer’s protocol. The quantitative real-time polymerase chain reaction (qRT-PCR) was performed using the ABI Prism 7500HT sequence detection system (Applied Biosystems, Darmstadt, Germany) with an SYBR green PCR master mix from Qiagen (Hilden, Germany). We investigated the genes encoding the mRNAs for IL-1β, TNF-α, IFNγ, and iNOS. All the primer assays used for the qRT-PCR were obtained commercially from Qiagen. The PCRs were run on the following schedule: 2 min at 50 °C to activate the uracil-N-glycosylase (UNG); 95 °C for 10 min to deactivate UNG; and then 40 cycles at 94 °C for 15 s, 60 °C for 35 s, and 72 °C for 30 s. The reaction specificity was determined by constructing dissociation curves after the PCR while the mRNA levels were normalized to 18S rRNA. The fold induction of the mRNA expression following infection with *P. chabuadi* was determined using the 2 − ΔΔCT method [24].

### Statistical analysis

A one-way analysis of variance (ANOVA) was used, and the statistical comparisons between the groups were performed using Duncan’s test using the statistical package for the social sciences SPSS, version 17.0) software. A *P* ≤ 0.05 was considered significant for all the statistical analyses.

## Results

**Fig. 1:**
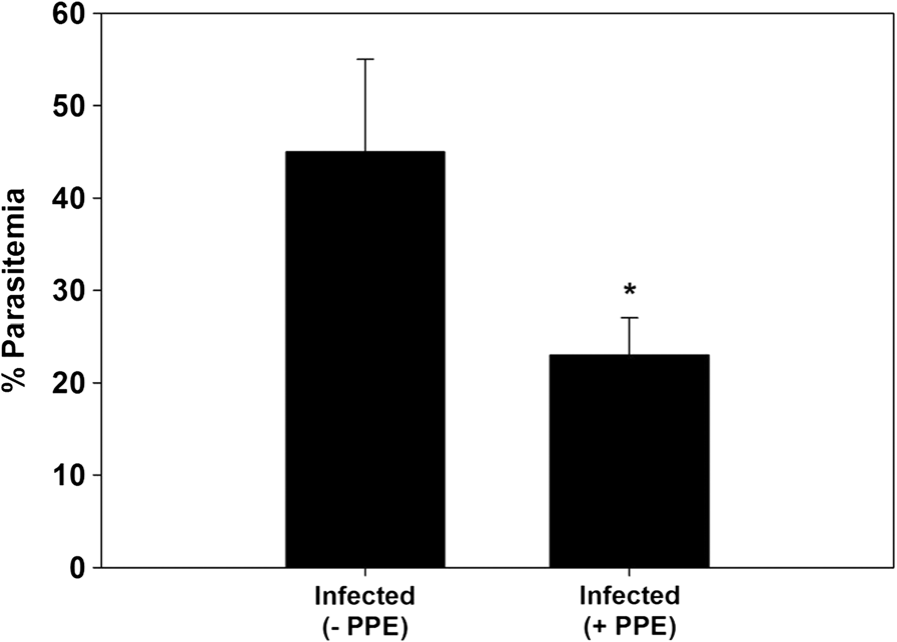
Effect of pomegranate peel extract (PPE) on parasitemia Bars represent two groups infected with *Plasmodium chabaudi*-parasitized erythrocytes, untreated (-PPE) and PPE-treated (+PPE) groups. Pomegranate significantly lowered percentage parasitemia on day 6 postinfection (p.i). Percentages are means of experimental duplicates. Error bars represent ± standard deviation (SD) of both groups. ^*^ *P* ≤ 0.05 compared with -PPE group

**Fig. 2:**
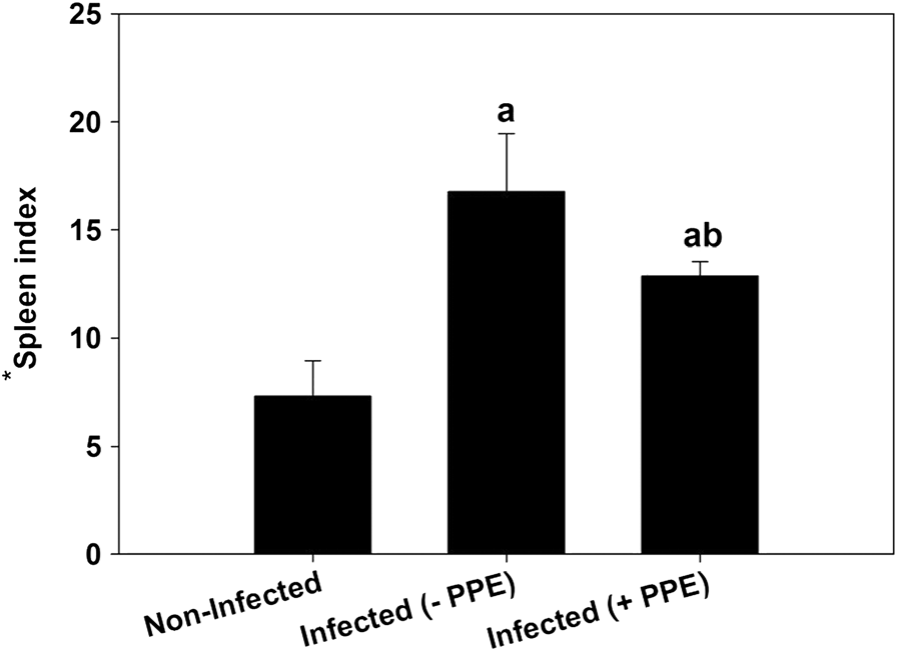
Pomegranate-induce changes in spleen index of mice infected with *Plasmodium chabaudi* parasitized erythrocytes Spleen index was calculated in non-infected (control), infected untreated (-PPE), and infected treated (+PPE) mice. ^a^ *P* ≤ 0.05 compared with non-infected control mice and ^ab^ *P* ≤ 0.05, comparing + PPE with –PPE groups. Spleen index was calculated as ratio of spleen weight (mg/mouse) to body weight (g/mouse)

**Fig. 3:**
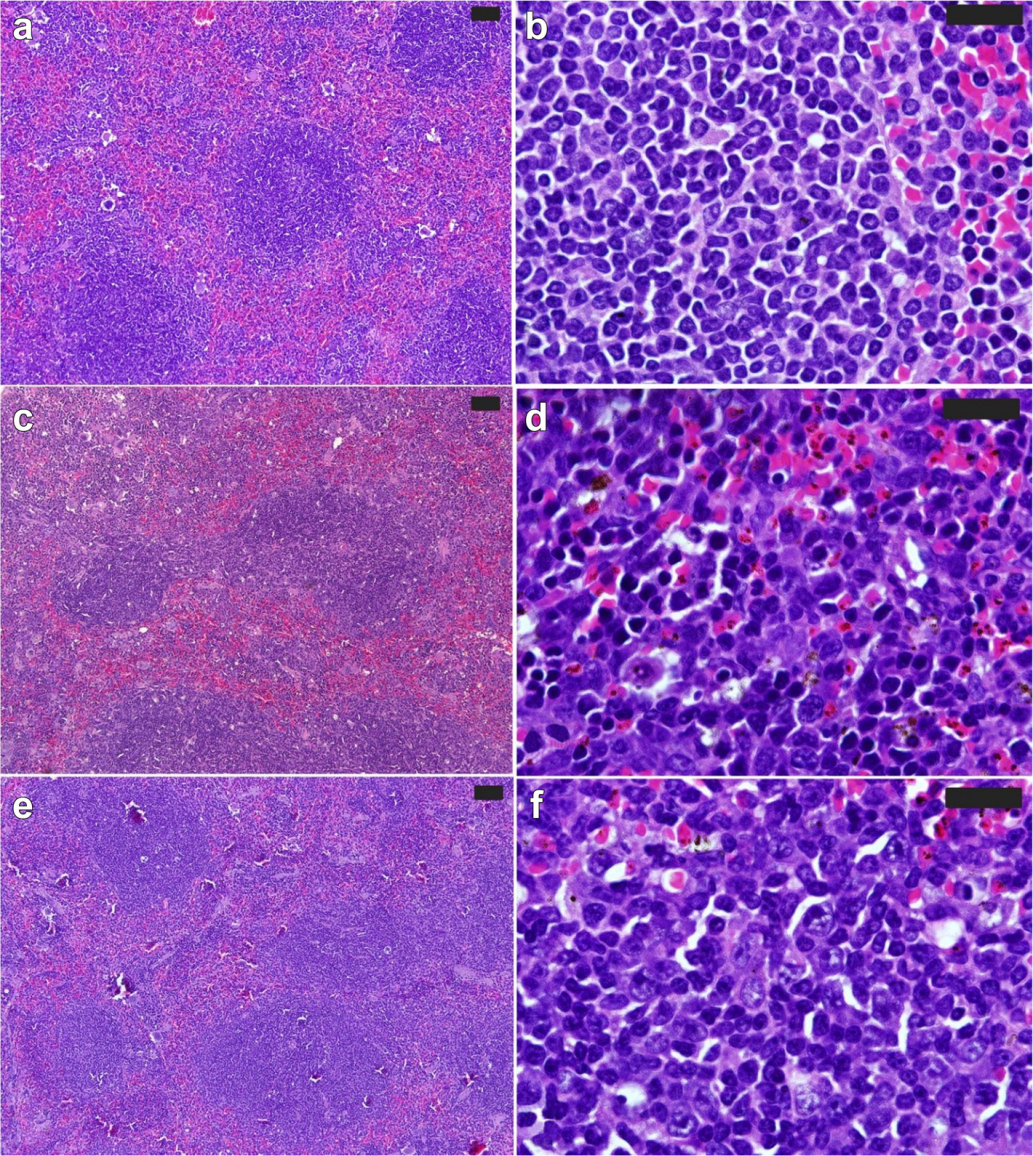
Pomegranate peel extract (PPE) improved spleen histopathological changes induced by *Plasmodium. chabaudi*-parasitized erythrocytes. **a** and **b** Non-infected spleen with normal architecture. **c** and **d** Infected spleen on day 6 post-infection (p.i.). White pulp (WP) is starting to fuse together. Spleen shows numerous hemozoin granules and infected erythrocytes in the red pulp (RP). Capsule (arrow head) of spleen appears thinner than that of control. **e** and **f** Infected mice treated with PPE. Spleens showed fewer lesion and improved tissue damage. Sections are hematoxylin and eosin-stained; scale bar = 25 μm

**Fig. 4:**
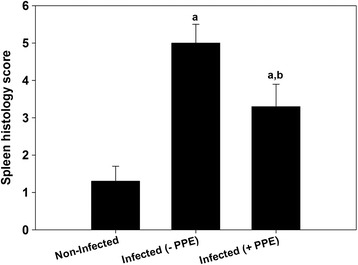
Pomegranate peel extract (PPE) induced spleen histology score changes in *Plasmodium chabaudi*-infected mice Enlarged spleen pulp areas are scored as (0, absent; 1, slight; 2, moderate; and 3, pronounced) *P. chabaudi* parasitized erythrocytes. Values are means ± standard deviation (SD). ^a^ *P* ≤ 0.05 compared with non-infected control mice. ^a,b^ *P* ≤ 0.05 comparing untreated infected (-PPE) and PPE-treated infected (+PPE) mice. Tissue sample score is mean score of high microscopic power fields of five different sections

**Fig. 5:**
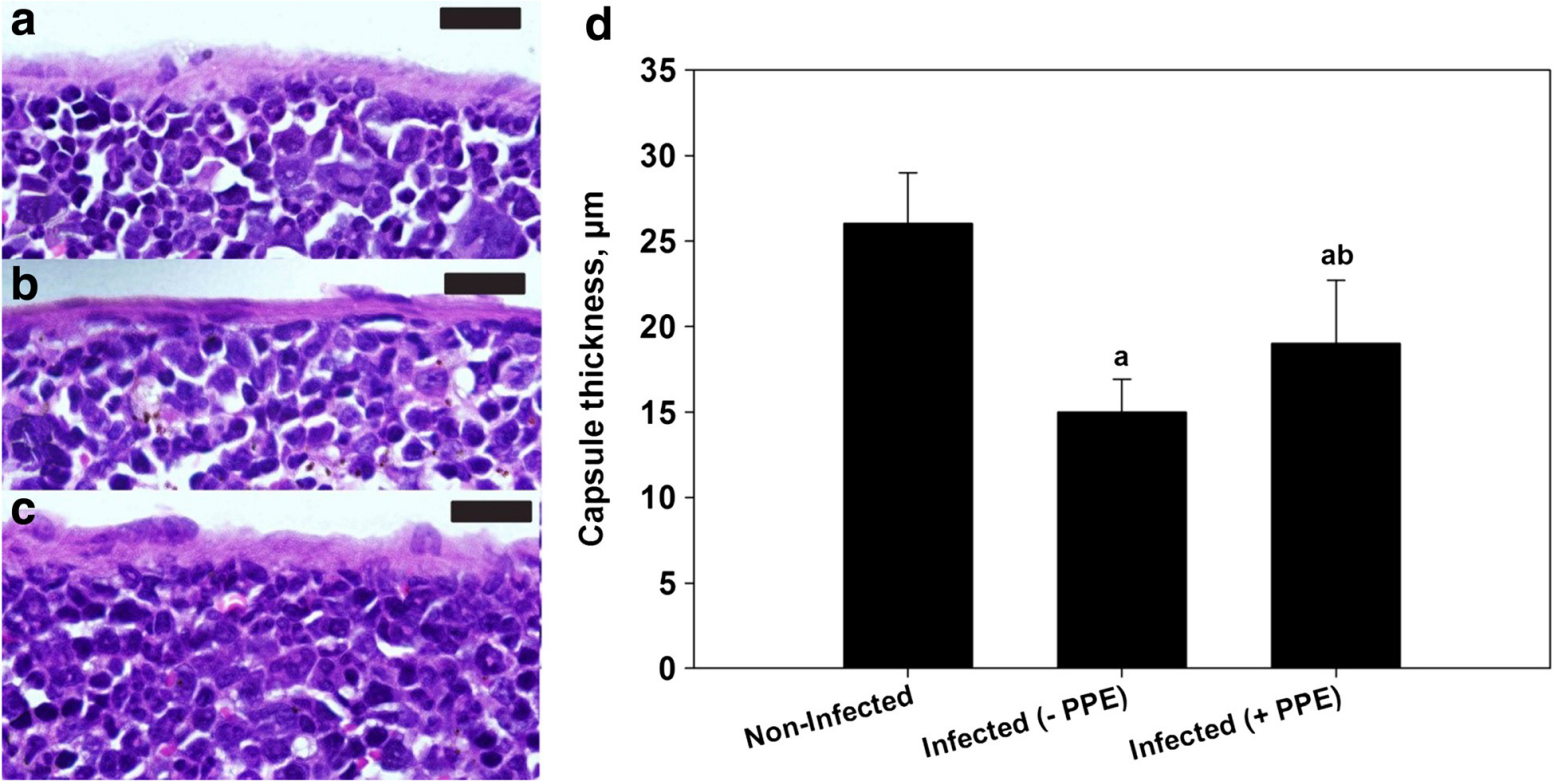
Spleen capsule thickness of uninfected and mice infected with *Plasmodium chabaudi*-parasitized erythrocytes. **a** Normal thickness of uninfected spleen capsule. **b** Infected spleen capsule on day 6 postinfection (p.i.). **c** PPE-treated infected mice show restoration of spleen capsule thickness. **d** Bar chart is thickness of spleen capsule (μm) among three groups, non-infected ~25 μm, PPE-treated infected mice, ~15 μm and PPE-treated infected mice with PPE ~20 μm. Each group represents an average of five different fields of spleen sections stained with hematoxylin and eosin; scale bar = 25 μm

The PPE-treated group of *P. chabaudi*-infected mice showed a significant reduction in the parasitemia percentage on day 6 p.i. to approximately 50 % of that of the untreated mice (Fig. 1). Moreover, the spleen index, which represents the spleen weight (mg/mouse) to body weight (g/mouse), remarkably increased in the untreated infected mice compared to that in the control (Fig. 2). However, interestingly, the spleen index of the PPE-treated mice decreased compared to that of the untreated mice (Fig. 2). This clearly indicates that the PPE effectively regulated the parasite and perhaps the clinical episodes. These findings were furthered supported by the improvement of the histopathological changes in the spleen of infected mice treated with PPE (Fig. 3). In addition, the histological scores showed that the enlargement of the spleen white pulp areas was significant in the *P. chabaudi*-infected mice compared to that in the uninfected group (Fig. 4). However, the PPE-treated group of infected mice showed a more significant amelioration than the untreated group did (Fig. 4). These results were supported by the evidence that the mice infected with *P. chabaudi*-pRBCs showed a reduction in the spleen capsule thickness compared to that of the uninfected control group (Fig. 5). In contrast, the PPE-treated group of infected mice exhibited a noticeable restoration of the spleen capsule thickness (Fig. 5).

Additionally, the spleens of the mice induced with *P. chabaudi* showed an upsurge in the levels of oxidant molecules (Table 1). However, the PPE attenuated the oxidative stress in the spleen of mice induced with *P. chabaudi*, demonstrating the antioxidant role of pomegranate (Table 1). On day 6 p.i., there was an increase in the levels of NO and MDA and a decrease in H_2_O_2_ level in the PPE-treated group compared to that of the control group (Table 1). In contrast, the PPE-treated group showed NO, MDA, and H_2_O_2_ levels that were almost comparable to those of the control. This clearly indicates that the *P. chabuadi* infection decreased the spleen catalase activity while it increased the NO and MDA activities, and these effects were inhibited by the PPE.

**Table 1:** Effect of pomegranate extract on spleen nitric oxide, malondialdehyde and catalase in mice infected with *P. chabaudi*

Groups	Nitric oxide (μmol/g)	Malondialdehyde (nmol/g)	Catalase (U/g)
Non-infected	424 ± 4	17.4 ± 1	17.1 ± 1
Infected (-PPE)	651 ± 14^ab^	23 ± 1^a^	7.6 ± 0.5^a^
Infected (+PPE)	438 ± 2^a^	12.4 ± 1^ab^	14.1 ± 0.6^ab^

Values are means ± SD (*n* = 6). ^a^Significant change at *p* < 0.05 with respect to non-infected mice

^ab^Significant change at *p* < 0.05 with respect infected mice

**Fig. 6:**
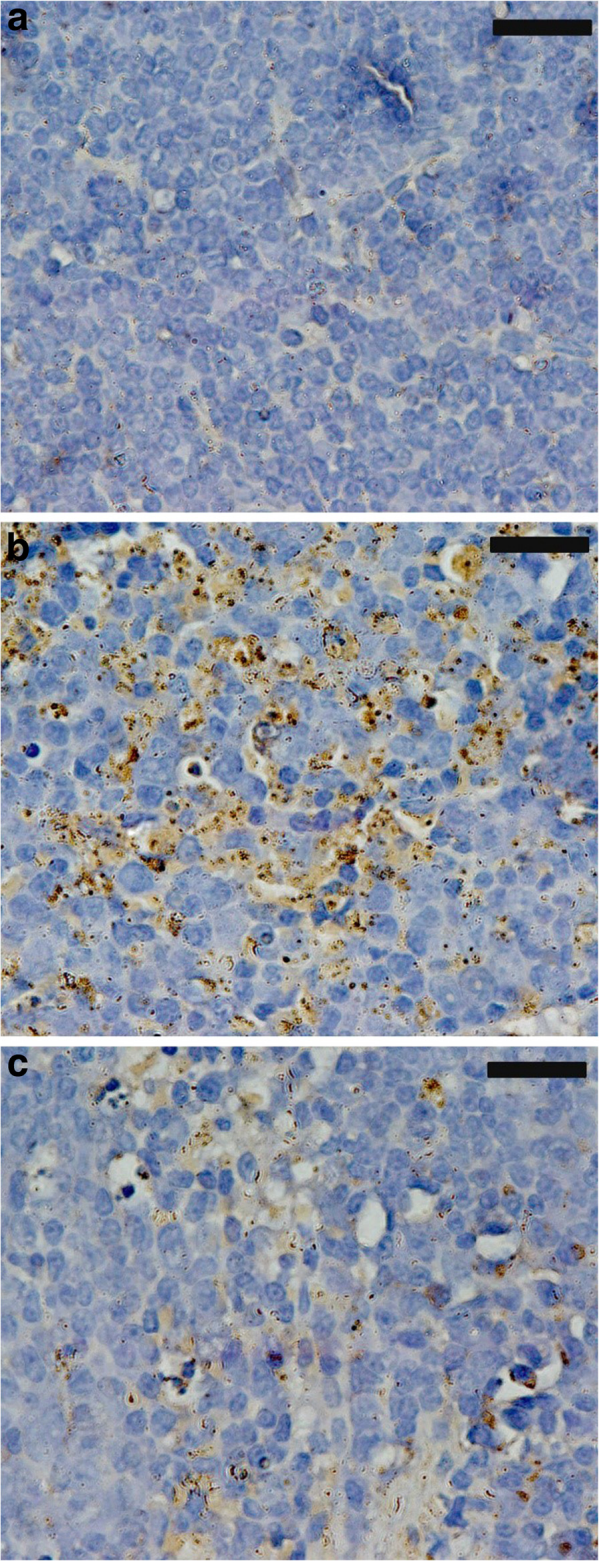
Immunohistochemical localization of terminal deoxynucleotidyl transferase (TdT) dUTP nick-end labeling (TUNEL)-positive cells in mouse spleens. **a** Uninfected spleen. **b** *Plasmodium chabaudi-*infected spleen with increased number of apoptotic cells. **c** Infected-pomegranate treated mice with decreased number of TUNEL-positive cells; scale bar = 25 μm

**Fig. 7:**
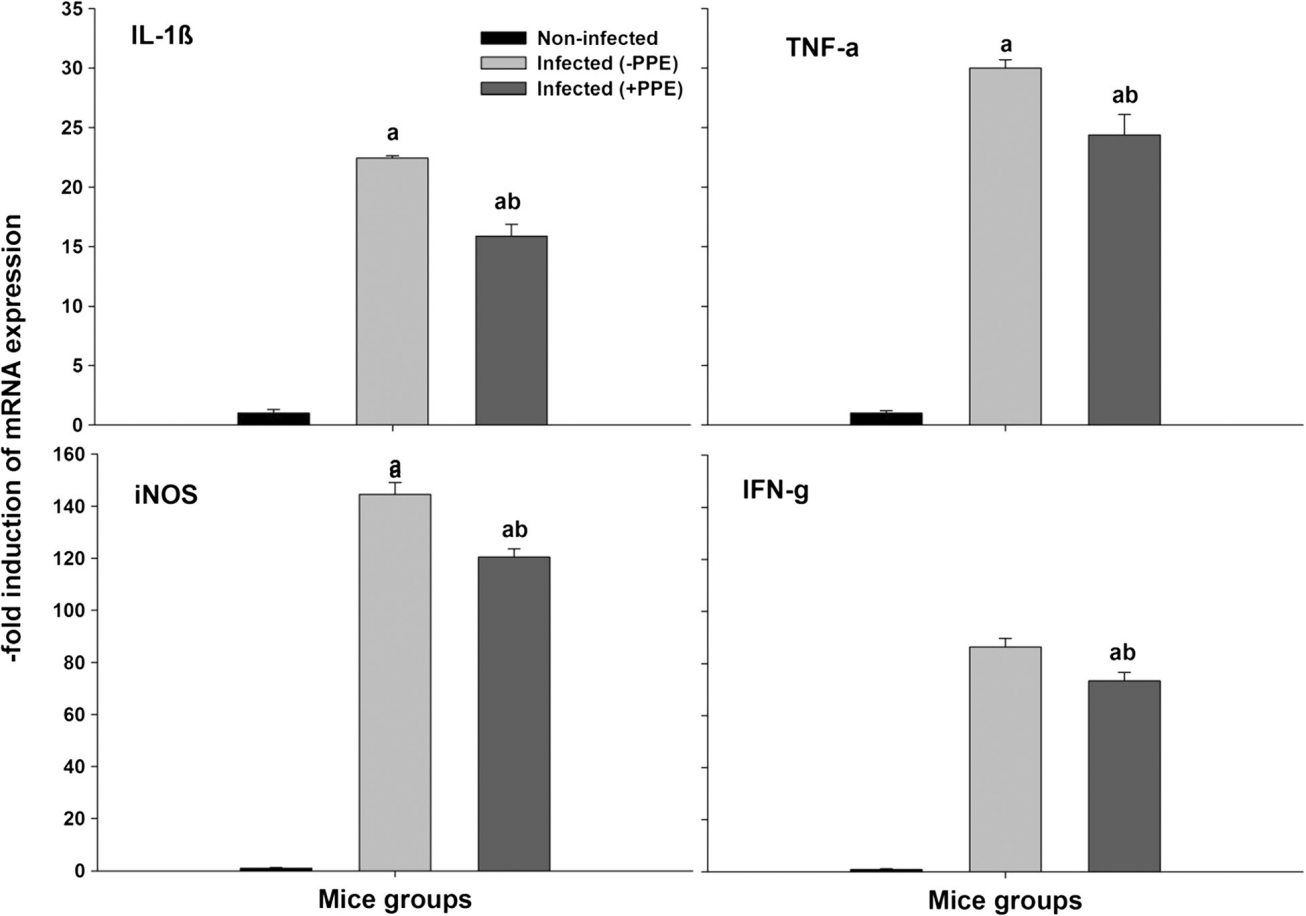
Quantitative real-time polymerase chain reaction (PCR) of mRNAs of proinflammatory cytokines Interleukin-(IL)-1β, tumor necrosis factor (TNF)-α, inducible nitric oxide synthase (iNOS), and interferon (IFN)-γ. Relative expression is fold increase compared with noninfected control mice. Values are means ± standard deviation (SD). ^a^ *P* ≤ 0.05 compared with uninfected control mice. ^ab^ *P* ≤ 0.05 comparing untreated infected (-PPE) and PPE-treated infected mice (+PPE)

Furthermore, Fig. 6 shows the histochemical alterations in the apoptotic spleen cells, and PPE reduced the number of TUNEL-positive spleen cells infected with *P. chabuadi* pRBCs. To assess the levels of proinflammatory cytokines, a qPCR analysis of the genes encoding the mRNA of IL-1β, TNF-α, iNOS, and IFNγ was conducted. Figure 7 shows that the mRNA levels of IL-1β, TNF-α, iNOS, and IFNγ were upregulated following the infection with *P. chabuadi* parasites compared to the levels of the uninfected mice. Conversely, there was a significant reduction in the mRNA levels of IL-1β, TNF-α, iNOS, and IFNγ of the mice following PPE treatment compared to the untreated controls (Fig. 7).

## Discussion

Malaria is a disease that causes millions of clinical episodes annually. Therefore, the discovery and development of preventive, therapeutic agents are considered necessary to control and manage the disease. While research in this area has achieved considerable progress, the problem of emerging parasite drug-resistant has also created additional challenges, making the mission more difficult. The beneficial role of medicinal plants in regulating malaria parasite infections has recently been shown [25–27]. Furthermore, malaria infection is associated with the release of proinflammatory cytokines including IL-1β, TNF-α, iNOS, and IFNγ, which play vital roles in mediating the severity of the disease [28]. Moreover, these proinflammatory cytokines are implicated in the pathogenesis and immunopathological reactions of the host-parasite interaction. Here, we propose that *P. granatum* treatment has beneficial effects on murine malaria-induced splenic injury and oxidative stress. For centuries, pomegranate has been considered as one of the candidate plants that shows potential therapeutic effects against numerous ailments as documented in different cultures [29]. The ameliorative effect of PPE in mice infected with the *P. chabaudi* parasite was investigated in this study. Mice infected with the *P. chabaudi* parasite achieved maximal parasitemia on day 6 p.i. during which the parasite induced the splenic responses. However, our findings showed that the parasitemia level decreased by 50 % following treatment with PPE. This observation illustrated the efficacy of PPE treatment compared to vehicle treatment and was in agreement with the results of our previous study [25].

Although malaria infection is characterized by splenic rupture and splenomegaly, the spleen is known as a key organ in the immune response development, and it senses infected RBCs [3, 4]. To evaluate the effect of PPE on the spleen, the thickness of the spleen capsules of uninfected mice, as well as those that were infected with or without treatment, was examined. The findings confirmed the beneficial effect of PPE, which conspicuously restored the spleen capsule thickness compared to that of the untreated group. This observation indicates that pomegranate inhibited the development of the *P. chabuadi* parasite in the host and perhaps acted by reducing the splenic inflammation. This suggests that the protective effect of PPE involves diminishing the oxidative destruction.

This is in accordance with the data of different studies that show that pomegranate peel and its biological properties are principally associated with the presence of flavonoids and tannins, and pomegranates have higher antioxidant properties than other fruits do [30–33]. Moreover, it has been reported that PPE reduces the production of NO and MDA while hindering the infection-induced loss of catalase activity [16]. Our data supported this notion by revealing that the supplementation of PPE to infected mice induced a superior recovery response from the oxidative stress-associated metabolites including H_2_O_2_, NO, and MDA compared to that of the untreated mice.

Furthermore, the modulatory effect of PPE on splenic injury and oxidative stress was also shown to be associated with the proinflammatory cytokines, IL-1β, TNF-α, iNOS, and IFNγ. The effects of PPE on the expression levels of IL-1β, TNF-α, iNOS, and IFNγ mRNA were investigated, and we obtained remarkable results. The results showed an upregulation of the mRNA levels of the proinflammatory cytokines in the untreated infected group of mice compared to the controls. However, the PPE-treated group of infected mice showed significantly downregulated mRNA levels compared to the untreated mice.

The cellular immune response arm of the adaptive immunity is known to degrade the pRBC by activating intracellular cytotoxicity mechanisms and IFNγ plays a key role in this process. Nonetheless, the increased level of IFNγ expression stimulates the responses of the local Th1/Th2 cell, which favor Th1 [6, 7]. In addition, IL-12 has been shown to be a proinflammatory Th1 cytokine that is promoted by the upregulation of IFNγ expression [34].

These findings suggest that PPE has anti-inflammatory activity and attenuates the inflammatory response of the innate immunity. Similar findings were obtained by Dkhil et al. [35] who reported the anti-inflammatory effect of berberine in mice infected with *Eimeria papillata*.

## Conclusion

In summary, this study describes the beneficial effects of pomegranate on splenic injury and oxidative stress in mice infected with *P. chabuadi*. Collectively, our data revealed the potential efficacy of pomegranate as an agent with antimalarial, antioxidant, and anti-inflammatory activities. The observed effects indicate that pomegranate protected the host mouse spleens and possibly other vital organs from damage induced by the *P. chabaudi* parasite. However, additional studies are required to further elucidate the exact underlying action mechanisms of pomegranate associated with the host-parasite interaction.

## Abbreviations

ANOVA, one-way analysis of variance; H_2_O_2_, hydrogen peroxide; HCl, hydrochloride; IFG, interferon; IL, interleukin; iNOS, inducible nitric oxide synthase; KSU, King Saud University; MDA, malondialdehyde; NO, nitric oxide; NPST, National Program for Science and Technology; PPE, pomegranate peel extract; pRBCs, parasitized red blood cells; qRT-PCR, quantitative real-time polymerase chain reaction; RBCs, red blood cells; TdT, terminal deoxynucleotidyl transferase; TNF, tumor necrosis factor; TUNEL, terminal deoxynucleotidyl transferase (TdT) dUTP nick-end labeling; UNG, uracil-N-glycosylase
